# Elaboration and validation of a prognostic signature associated with disulfidoptosis in lung adenocarcinoma, consolidated with integration of single-cell RNA sequencing and bulk RNA sequencing techniques

**DOI:** 10.3389/fimmu.2023.1278496

**Published:** 2023-10-27

**Authors:** Dabao He, Hengfeng Tang, Xiaoling Yang, Xiaohong Liu, Yipeng Zhang, Junzhu Shi

**Affiliations:** ^1^ Department of Laboratory Medicine, Shenzhen Longhua District Central Hospital, Shenzhen, China; ^2^ Department of Laboratory Medicine, Shenzhen Baoan District Songgang People’s Hospital, Shenzhen, China; ^3^ Department of Oncology, Shenzhen Longhua District Central Hospital, Shenzhen, China

**Keywords:** lung adenocarcinoma, single-cell analysis, endothelial cells, disulfidptosis, prognostic risk signature, immune infiltration, drug prediction

## Abstract

**Background:**

Lung adenocarcinoma (LUAD), the predominant subtype of non-small cell lung cancer (NSCLC), remains a pervasive global public health concern. Disulfidoptosis, a nascent form of regulated cell death (RCD), presents an emerging field of inquiry. Currently, investigations into disulfidoptosis are in their initial stages. Our undertaking sought to integrate single-cell RNA sequencing (scRNA-seq) in conjunction with traditional bulk RNA sequencing (bulk RNA-seq) methodologies, with the objective of delineating genes associated with disulfidoptosis and subsequently prognosticating the clinical outcomes of LUAD patients.

**Methods:**

Initially, we conducted an in-depth examination of the cellular composition disparities existing between LUAD and normal samples using scRNA-seq data sourced from GSE149655. Simultaneously, we scrutinized the expression patterns of disulfidoptosis-associated gene sets across diverse cell types. Subsequently, leveraging the bulk RNA-seq data, we formulated disulfidoptosis-related prognostic risk signatures (DRPS) employing LASSO-Cox regression. This was accomplished by focusing on genes implicated in disulfidoptosis that exhibited differential expression within endothelial cells (ECs). Sequentially, the robustness and precision of the DRPS model were rigorously verified through both internal and external validation datasets. In parallel, we executed single-cell trajectory analysis to delve into the differentiation dynamics of ECs. Concluding our study, we undertook a comprehensive investigation encompassing various facets. These included comparative assessments of enrichment pathways, clinicopathological parameters, immune cell abundance, immune response-associated genes, impacts of immunotherapy, and drug predictions among distinct risk cohorts.

**Results:**

The scrutiny of scRNA-seq data underscored discernible disparities in cellular composition between LUAD and normal samples. Furthermore, disulfidoptosis-associated genes exhibited marked discrepancies within endothelial cells (ECs). Consequently, we formulated the Disulfidoptosis-Related Prognostic Signature (DRPS) to facilitate prognostic prediction. The prognostic nomogram based on the risk score effectively demonstrated DRPS’s robust capacity to prognosticate survival outcomes. This assertion was corroborated by rigorous assessments utilizing both internal and external validation sets, thus affirming the commendable predictive accuracy and enduring stability of DRPS. Functional enrichment analysis shed light on the significant correlation of DRPS with pathways intrinsic to the cell cycle. Subsequent analysis unveiled correlations between DRPS and gene mutations characteristic of LUAD, as well as indications of an immunosuppressive status. Through drug prediction, we explored potential therapeutic agents for low-risk patients. Concluding our investigation, qRT-PCR experiments confirmed the heightened expression levels of EPHX1, LDHA, SHC1, MYO6, and TLE1 in lung cancer cell lines.

## 1 Introduction

Lung adenocarcinoma (LUAD), a distinct subset within the non-small cell lung cancer (NSCLC) classification, currently stands as the most prevalent manifestation of lung malignancy, exhibiting a gradual upward trajectory in its prevalence in recent years (1, 2). Contemporary therapeutic approaches for managing LUAD encompass an array of modalities such as surgical intervention, chemotherapy, immunotherapy, and targeted therapy, deployed either singularly or in tandem contingent upon tumor characteristics (3). Nevertheless, the inconspicuous onset characteristic of LUAD often leads to delayed detection, consequently missing the critical window for prompt diagnosis and intervention (4). The paucity of requisite biomarkers for early-stage detection remains a formidable hurdle in the clinical diagnosis and therapeutic regimen of LUAD. Furthermore, the marked aggressiveness, drug resistance, proclivity for relapse, and evolvement of immune resistance collectively underpin the challenging nature of LUAD. Notwithstanding the remarkable strides attained in the clinical application of innovative interventions such as immunotherapy and targeted therapy, a substantial majority of advanced-stage LUAD patients continue to face a dire prognosis, with survival spans rarely extending beyond the 5-year mark (5, 6). Consequently, the identification of novel, exquisitely responsive biomarkers or therapeutic targets emerges as an imperative. This pursuit holds the potential to foster tailored treatment strategies, thereby mitigating the adverse effects associated with treatment regimens and potentially enhancing clinical efficacy.

Disulfidoptosis represents a newly characterized variant of regulated cell death (RCD), incited by the aberrant intracellular buildup of disulfides. (7). From a mechanistic standpoint, when cells experience glucose deprivation, the elevated expression of solute carrier family 7 member 11 induces a reduction in cytoplasmic levels of nicotinamide adenine dinucleotide phosphate. Consequently, this depletion leads to the accumulation of irreducible intracellular disulfide compounds, which subsequently initiates the formation of disulfide bonds between actin cytoskeletal proteins. Ultimately, this process results in the collapse of the actin filament network and ultimately triggers disulfide ptosis. (8). The distinctive morphological characteristics exhibited by disulfiprosis, which induce cellular demise through the alteration of cytoskeletal protein conformation, facilitate its clear differentiation from alternative types of regulated cell death (RCD) such as ferroptosis and copper-induced cell death (9, 10). Disulfidoptosis not only establishes a linkage between cellular metabolism and cellular destiny but also demonstrates a conspicuous association with the immune response within the tumor microenvironment (11, 12). Currently, inducing disulfidptosis of tumor cells is considered a promising therapeutic strategy. The investigation into disulfidptosis is still in its early stages, particularly with limited research on disulfidptosis and LUAD. Despite the availability of solely conventional bulk RNA sequencing (bulk RNA-seq) data, there is an opportunity to investigate the potential prognostic indicators of LUAD. For example, Cui Qi et al. Developed a predictive risk model using bulk RNA-seq data to illustrate the possible correlation between genes associated with disulfidptosis and the progression of lung adenocarcinoma (11). Nevertheless, LUAD is an intricately intricate and diverse solid neoplasm consisting of various cellular populations. The exclusive reliance on bulk RNA-seq for analysis will mask the precise details of individual cells, particularly in cases of LUAD with intricate heterogeneity (13). This problem has been addressed by the advent of scRNA-seq, a technique for sequencing RNA at the single-cell level. The single-cell RNA sequencing has offered unexpected fresh perspectives on the associated investigations of cancer development, tumor diversity, and tumor surroundings (14).

To commence, we embarked on an examination of the compositional constitution of lung adenocarcinoma (LUAD) utilizing a scRNA-seq dataset (GSE149655) sourced from the gene expression omnibus (GEO). The aim was to elucidate the inherent advantages conferred by single-cell sequencing techniques. Additionally, we scrutinized the prevalence of disulfidoptosis-related genes across diverse cell types for enrichment rationale. Pronounced disparities emerged in the expression profiles of genes implicated in disulfidoptosis within endothelial cells (ECs), discernibly demarcating tumor tissues from their normal counterparts. In parallel, capitalizing on the bulk RNA-seq dataset (TCGA-LUAD) procured from the cancer genome atlas (TCGA), we effectively identified a collection of nine genes displaying differential expression (DEGs) via the employment of LASSO-Cox regression. Subsequent to this, we adeptly formulated a prognostic signature (DRPS) intimately linked to the disulfidoptosis phenomenon. Extensive scrutiny ensued to delineate the nexus between DRPS and a spectrum of clinicopathological attributes, alongside the overarching survival outcomes (OS) among LUAD patients. Additionally, we delved into the mutation status of the nine genes implicated in disulfidoptosis within the context of LUAD. Validation of the prognostic risk signature was systematically executed across both internal and external validation cohorts. Furthermore, an auxiliary exploration was conducted to unveil the interplay between DRPS and the responsiveness to immunotherapy interventions. Our comprehensive research endeavor furnishes an enriched comprehension of disulfidoptosis-associated genes as potential harbors of prognostic insights and therapeutic targets within the LUAD landscape. In this vein, our findings present novel dimensions to the evaluation and strategic management of LUAD.

## 2 Materials and methods

### 2.1 Dataset collection and preprocessing

The single-cell RNA sequencing dataset (GSE149655) was obtained from the Gene Expression Omnibus (GEO) repository. From the TCGA and GEO databases, we acquired a large RNA-seq dataset (TCGA-LUAD, GSE68465, GSE50081, GSE37745, GSE31210, and GSE3141) specifically for LUAD. Samples without T stage, N stage, M stage, and clinical stage information were removed, as well as samples marked with survival time = 0. Thus we acquired 500 samples from TCGA-LUAD, 442 samples from GSE68465, 127 samples from GSE50081, 226 samples from GSE37745, 106 samples from GSE31210, and 111 samples from GSE3141. To confirm the association between prognostic characteristics and response to immune checkpoint inhibitors (ICIs), the IMvigor210 datasets were employed as validation sets. Gene read count values were also normalized for these patients. Publicly available data includes TCGA, GEO, and IMvigor210 datasets.

### 2.2 Single-cell sequencing data processing

To analyze single-cell transcriptomics data, we utilized the Seurat R package (v4.1.0). The function of Seurat, “NormalizeData” were used to normalize the counts of single-cell transcriptomics. The filter conditions were set as: 500 ≤ nCount_RNA ≤ 4000, 400 ≤ nFeature_RNA ≤ 10000, percent.mt ≤ 15, percent. Ribo ≤ 20. Lognormalize method was used to normalize the data. The dimensionality was reduced and the main cell clusters were found using the methods ‘RunPCA’, ‘FindNeighbors’, and ‘FindClusters’. The clustering parameter was set as 1.2. Subsequently, UMAP, a method called Uniform Manifold Approximation and Projection, is utilized to reduce the dimensionality of datasets and represent them in a lower-dimensional space (typically two or three dimensions) for visualization purposes. The annotation of single-cell RNA sequencing data obtained from the Gene Expression Omnibus (GEO) database was utilized to annotate the clusters of cells in the dataset GSE149655. For each cluster, the FindAllMarkers function was utilized to detect (DEGs) with a logic. threshold of 0.25. Identification of cell types was conducted by utilizing the differentially expressed genes (DEGs) within each cluster and subsequently verified through manual examination, as outlined in a prior investigation.

### 2.3 Score according to the disulfidptosis-related gene set

We curated a collection of 32 disulfidoptosis-related genes, integral to cancer research, constituting the disulfidoptosis-related gene set. Leveraging the GSE149655 dataset, we harnessed algorithms including AUCell, Ucell, singscore, ssGSEA, and AddModuleScore (Add) to compute gene set scores for the disulfidoptosis-related gene sets (15, 16). Subsequently, the outcomes of these five gene set scores were amalgamated and standardized for the purpose of Scoring analysis. Differences pertaining to six distinct cell types across tumor and normal tissues were visually presented through the utilization of the ggplot2 R package (version 3.3.5).

### 2.4 Intercellular communication

To elucidate the intercellular interaction networks, we employed the CellChat tool (version 1.1.3). Specifically, we employed the ‘AggregateNet’ feature of CellChat, which integrates diverse cellular communication networks. These networks encompass various cell types, including epithelial cells, NK cells, T cells, macrophages, endothelial cells (ECs), and smooth muscle cells.To examine the signaling pathways within the intercellular communication network, we employed the “netVisual_aggregate” function provided by CellChat. This function allowed us to compute and visualize the inherent signaling pathways. Furthermore, to determine the significance of specific cell types within the tissue microenvironment, we used the “netAnalysis_computeCentrality” function in CellChat. This function enabled us to calculate centrality scores, providing insights into the predominant signaling roles of distinct cell types.

### 2.5 Creation and verification of a prognostic risk signature

The test set includes the TCGA-LUAD dataset. Internal validation sets encompass GSE68465, GSE5008, GSE3774, GSE31210, and GSE3141, while IMvigor210 serves as an external validation set. These datasets collectively underpin our model’s assessment. The investigation dataset aids in identifying disulfidoptosis-associated genes and crafting prognostic risk signatures. To prevent overfitting, we employ the LASSO penalized Cox proportional hazards regression technique via the glmnet R package. Our signature is determined using a penalty parameter (λ), assessed through ten-fold cross-validation against stringent criteria. We compute patient risk scores using gene expression levels and their corresponding coefficients. These scores are the sum of gene expression values multiplied by their coefficients (β). Patients are categorized into high- or low-risk groups based on median risk scores. We then conduct a Kaplan-Meier analysis to compare overall survival curves between these groups. Additionally, we employ time-dependent ROC analysis to illustrate survival rates at 1, 3, and 5 years. The Area Under the Curve (AUC) quantifies the signature’s predictive ability.

### 2.6 Univariate and multivariate Cox regression analysis

Cox regression analysis was performed to ascertain whether the risk score held standalone prognostic significance. Univariate Cox regression analysis encompassed variables including age, gender, tumor grade, TNM stage, and risk score. Significant factors thus identified were subsequently integrated into the multivariate Cox regression analysis, adjusting for potential confounders and covariates to assess the independent prognostic significance of each variable. To summarize the outcomes, we presented the results in a forest plot, which provides a concise overview of the impact of each variable on patient prognosis.

### 2.7 Analyses of clinical correlations

Clinical correlation analysis was undertaken across diverse patient cohorts to glean insights into the interplay between the risk score and clinical characteristics. For the assessment of attributes’ sensitivity and specificity, the survivalROC R package was harnessed to construct ROC curves and ascertain the associated AUC values. Capitalizing on both the risk score and clinical attributes, a nomogram for LUAD was meticulously formulated using the ‘rms’ script.

### 2.8 Correlation between DRPS and clinicopathological features

Clinical correlation analysis was conducted on various patient groups to enhance comprehension of the association between DRPS and clinical characteristics. Moreover, the integration of DRPS with other clinical parameters and biomarkers may be necessary for enhancing predictive accuracy and clinical applicability in the context of its implementation in clinical practice.

### 2.9 Functional enrichment analysis

Utilizing the limma R package, we extracted the differentially expressed genes (DEGs) within the TCGA-LUAD dataset by contrasting the high-risk and low-risk groups. To evaluate the functional enrichment of these DEGs, we carried out stepwise analyses involving both Gene Ontology (GO) and Kyoto Encyclopedia of Genes and Genomes (KEGG) databases. Furthermore, we subjected the enrichment outcomes of the nine prognostic genes to gene set variation analysis (GSVA). To this end, we accessed the h.all.v7.1.symbols Oncogenic Signature Pathway Gene Set, sourced from the MSigDB database.

### 2.10 Gene mutation and DNA damage repair

We utilized the maftools R package to examine nine genes associated with prognosis using TCGA’s somatic mutation data (PMC5982584). We explored the impact of these nine genes on oncogenic pathways, copy number variation (CNV) status, and associations between DNA damage measures and survival risk scores. In addition, we analyzed mutual co-occurrence or exclusive mutations among the top 19 mutated genes. atlas provides a comprehensive examination of DDR deficiencies in various types of cancer (17). We utilized this source to investigate various markers of genetic instability, such as the rate of mutations that cause changes in protein coding, count of genomic segments, proportion of altered genetic material, deficiencies in homologous recombination, and a score indicating abnormal chromosome numbers.

### 2.11 Immune status assessment and immune microenvironment analysis

The ESTIMATE algorithm was employed to evaluate the infiltration of stromal and immune microenvironment based on gene transcriptome information. Using the TCGA-LUAD dataset, we employed the CIBERSORT algorithm to examine the immune infiltration of tumors in the high- and low-risk groups. Additionally, we utilized the ESTIMATE algorithm to assess the proportion of immune stromal components in the high- and low-risk groups. We investigated the expression of inhibitory receptors and ligands of immune cells in high- and low-risk groups using the Spearmen’s correlation test. Using the TIDE algorithm, we evaluated the potential for tumor immune escape by analyzing the gene expression profiles of both high- and low-risk groups.

### 2.12 Immune response analysis

The IMvigor210 datasets were used as external validation sets for the correlation between prognostic features and treatment response to ICIs, including differences in efficacy and survival outcomes. The effectiveness of ICIs was assessed based on the Response Evaluation Criteria in Solid Tumors (RECIST) guidelines. Patients who showed complete response (CR) or partial response (PR) were referred to as responders, whereas patients with stable disease (SD) or progressive disease (PD) were classified as non-responders.

### 2.13 Single-cell trajectory analysis

We examined the trajectories of endothelial cells (ECs) within scRNA-seq data through the creation of pseudo-temporal developmental trajectories using Monocle2 (version 2.22.0) (13). The alterations in expression levels of prognostic genes along the pseudo-temporal developmental direction were visualized using heatmaps.

### 2.14 Drug prediction

In order to identify appropriate chemotherapy medications for low-risk patients, we conducted a thorough investigation utilizing the Cancer Genome Project (CGP) database. This database provided valuable information regarding various chemotherapy medications. We meticulously assessed the response to chemotherapy in both low-risk and high-risk patient groups. To further enhance our predictive capabilities, we leveraged the pRRophetic R package. Using this powerful computational tool, we accurately predicted the IC50 values of chemotherapeutic drugs for each individual patient. This gave us valuable insights into the potential efficacy of different chemotherapy treatments. Based on these comprehensive analyses, we recommended specific drugs or drug classes for low-risk patients.

### 2.15 Verification of DRPS

To confirm the levels of gene expression of DRPS in tumor and normal cells, we utilized quantitative real-time polymerase chain reaction (qRT-PCR). Procell (Wuhan Procell Life Science and Technology Co. Ltd., Wuhan, China) supplied two variants of non-small cell lung cancer cells (A549 and H1299) and one variant of normal lung epithelial cell (BEAS-2B). Every cell line was grown in its specific culture medium provided by Wuhan Procell Life Science and Technology Co. Ltd., located in Wuhan, China. The cells were grown in RPMI-1640 (Gibco-BRL) medium, which was enriched with 10% fetal bovine serum (Bioserum), 100 U/mL penicillin G, and 100 μg/mL streptomycin.AG RNAex Pro reagent (Accurate Biology) was used to extract total RNA from BEAS-2B, A549, and H1299. The reverse transcriptase reaction was performed using the Evo M-MLV RT Mix Tracking Kit from Accurate Biology, and detection was carried out using SYBR-Green, also from Accurate Biology. GAPDH was used to normalize the mRNA expression levels of TLE1, LDHA, SHC1, EMC6, HTATIP2, JAG1, EPHX1, MYO6, and HERPUD1. The LDHA primers used were ATGGCAACTCTAAAGGATCAGC (forward primer) and CCAACCCCAACAACTGTAATCT (reverse primer); the SHC1 primers used were GCCAAAGACCCTGTGAATCAG (forward primer) and GTATTGTTTGAAGCGCAACTCG (reverse primer); the EPHX1 primers used were CTTTGCCATCTACTGGTTCATCT (forward primer) and TCTCCTCATCTGACGTTTCCA (reverse primer); the MYO6 primers used were TATTGTGGATATTGGCCCCGA (forward primer) and TGGATTCACTGCAATCAGAATGT (reverse primer); the TLE1 primers used were GAGTCCCTGGACCGGATTAAA (forward primer) and AATACATCACATAGTGCCTCTGC (reverse  primer); and the GAPDH primers used were ACCCACTCCTCCACCTGA (forward primer) and TCCACCACCCTGTTGCTGTA (reverse primer).Calculate the fold difference for each group using normalized CT values.

### 2.16 LDHA knockdown and cell proliferation assay

Cell Counting Kit-8 was purchased from Med Chem Express, Cat. No.: HY-K0301. LDHA antibody was acquired from abcam, Cat. No.: ab300637. siLDHA was designed via http://sidirect2.rnai.jp/, with LDHA siRNA1: 21nt guide (5’→3’) UUAUCAGUCCCUAAAUCUGGG and 21nt passenger (5’→3’) CAGAUUUAGGGACUGAUAAAG; LDHA siRNA2: 21nt guide (5’→3’) UUCCUUAUCUUUAUCAGUCCC and 21nt passenger (5’→3’) GACUGAUAAAGAUAAGGAACAC.

### 2.17 Statistical analysis

All bioinformatic analyses were performed using R 4.0.3. Cox regression analysis was utilized to compare the K-M survival curve. The Wilcoxon rank sum test was used to compare the variation in expression levels between groups. Correlation analysis involved the utilization of Pearson correlation. Values with r greater than 0.1 were regarded as relevant, while those with P less than 0.05 were considered statistically significant. In this study, the symbols ‘*’, ‘**’, and ‘***’ were used to represent statistical significance levels of P < 0.05, P < 0.01, and P < 0.001, respectively.

## 3 Results

### 3.1 Single-cell sequencing analysis

**Figure 1:**
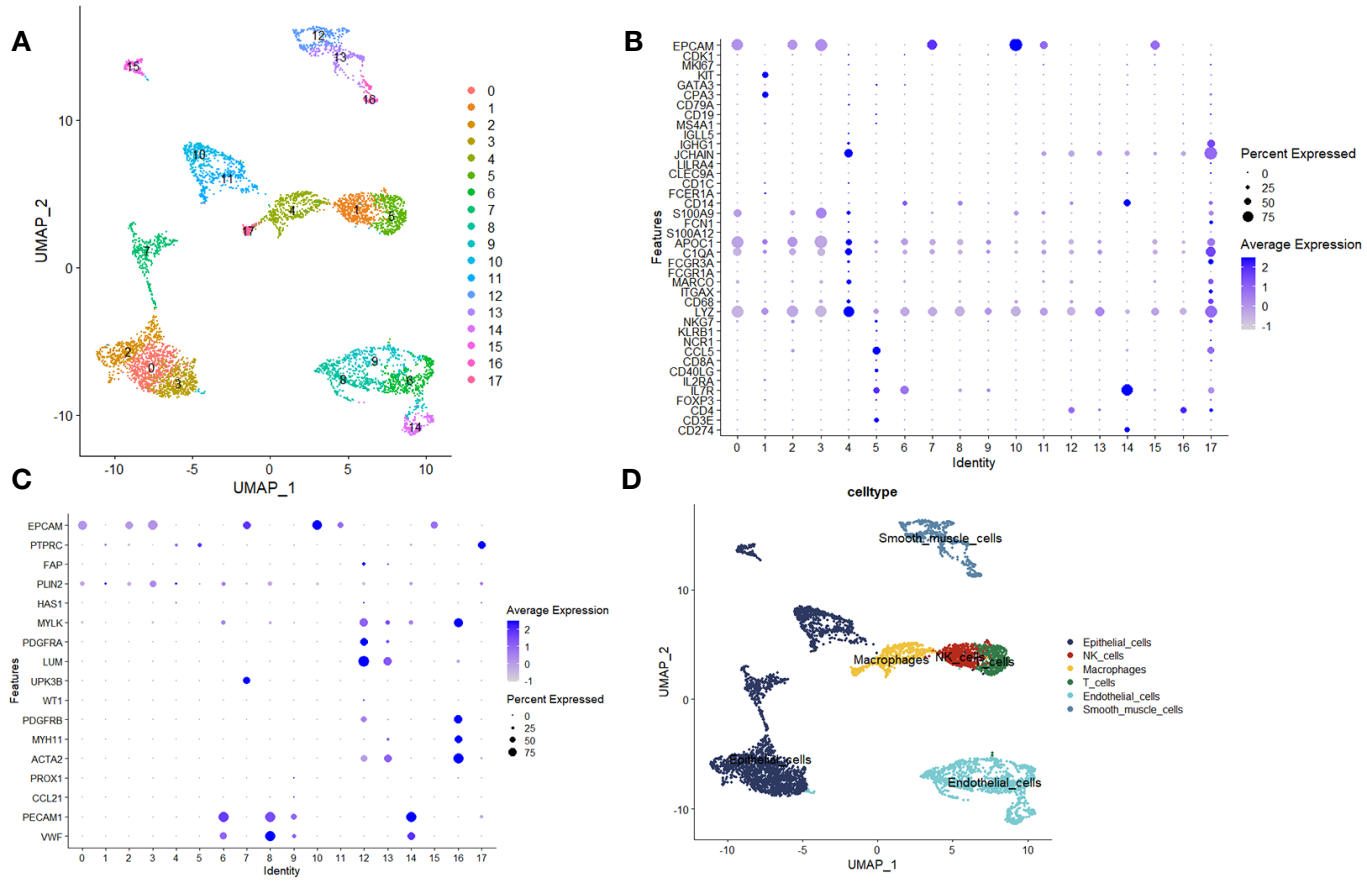
Analysis utilizing single-cell sequencing. **(A)** Unified Manifold Approximation and Projection (UMAP) depictions demonstrating the outcomes of dimensionality reduction and clustering. **(B, C)** Graphical representations illustrating the mean expression levels and relative prevalence of marker genes within the 17 discerned clusters. **(D)** UMAP plots elucidating the six distinct cell types, namely epithelial cells, Natural Killer (NK) cells, T cells, macrophages, endothelial cells (ECs), and smooth muscle cells.

Inceptionally, we scrutinized the disparities in composition between samples afflicted with Lung Adenocarcinoma (LUAD) and their corresponding healthy counterparts via scRNA-seq datasets. The dataset GSE149655 encompasses singular-cell sequencing data procured from tumor lesion tissue and its distal normal lung tissue counterpart, harvested from treatment-naïve LUAD patients. In the preliminary phase, we executed dimensionality reduction, clustering, and the visualization of GSE149655, employing the Seurat R package and the UMAP algorithm. This comprehensive analysis unveiled the discernment of 17 distinct clusters within the specimen (**Figure 1A**). These clusters were subsequently categorized into six distinct cell types, precisely: epithelial cells, Natural Killer (NK) cells, T cells, macrophages, endothelial cells (ECs), and smooth muscle cells. Such classification was founded upon the gene expression profiles inherently exhibited by the cells within each designated cluster (**Figures 1B–D**).

### 3.2 Cell scoring algorithm based on disulfidptosis-related genes

**Figure 2:**
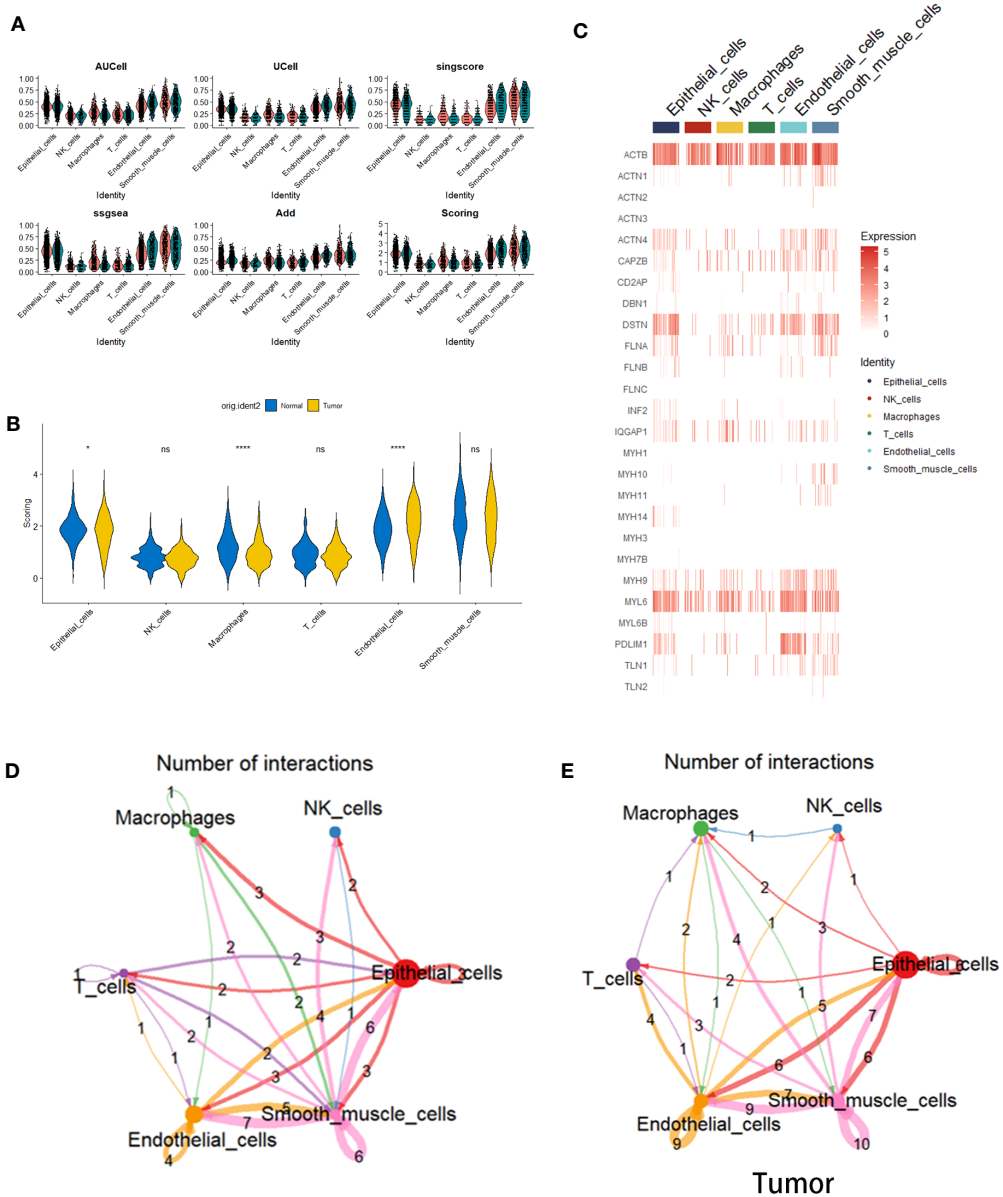
Cell scoring algorithm based on disulfidptosis-related genes. **(A)** Violin plots depicting the enrichment status of disulfidptosis-related gene sets within six distinct cell types situated in both tumor and normal tissues. This assessment is executed employing diverse gene set scoring algorithms. **(B)** Violin plots elucidating the amalgamated and standardized outcomes stemming from the utilization of the five gene set scoring algorithms. **(C)** A heatmap representation, visualizing the expression levels of genes linked with disulfidptosis across the spectrum of six cell types. **(D)** Violin plots illustrating the disparities within Scoring scores for gene sets correlated with disulfidptosis within the context of the six cell types, differentiating between tumor and normal tissues (*p< 0.05 and ****p<0.0001). **(E)** Depiction of a cell-cell interaction network. The colors of arrows and edges provide directional cues. The thickness of edges denotes the magnitude of interactions between cell groups. Loops signify autocrine circuits.

In order to scrutinize the potential correlation between these cellular entities and disulfide-mediated apoptosis (disulfidptosis), an assessment was conducted to evaluate the degree of enrichment pertaining to gene sets related to disulfidptosis across the aforementioned six distinct cell types. This evaluation was executed utilizing five distinct gene set scoring algorithms, namely AUCell, UCell, singscore, ssGSEA, and Add (**Figure 2A**). The subsequent phase involved the amalgamation and normalization of the aforementioned scoring outcomes, as visualized in **Figure 2B**. The heatmap representation facilitated the observation of the expression patterns of genes associated with disulfidptosis within the aforementioned six cell types (**Figure 2C**). Ultimately, discernment emerged, showcasing that gene sets correlated with disulfidptosis exhibited a notable enrichment pattern within epithelial cells and endothelial cells (ECs) situated within tumor tissues, alongside macrophages present within normal tissues (**Figure 2D**). It is noteworthy to emphasize the pivotal role assumed by ECs in the context of tumor development (18, 19). Further investigations, utilizing the CellChat tool, unearthed substantial disparities inherent in the intercellular communication network between normal and tumor tissues (**Figure 2E**). These findings pointed towards noteworthy alterations in both the strength of interactions and the composition of cell types involved.

### 3.3 Validation and creation of a prognostic signature associated with disulfidptosis

**Figure 3:**
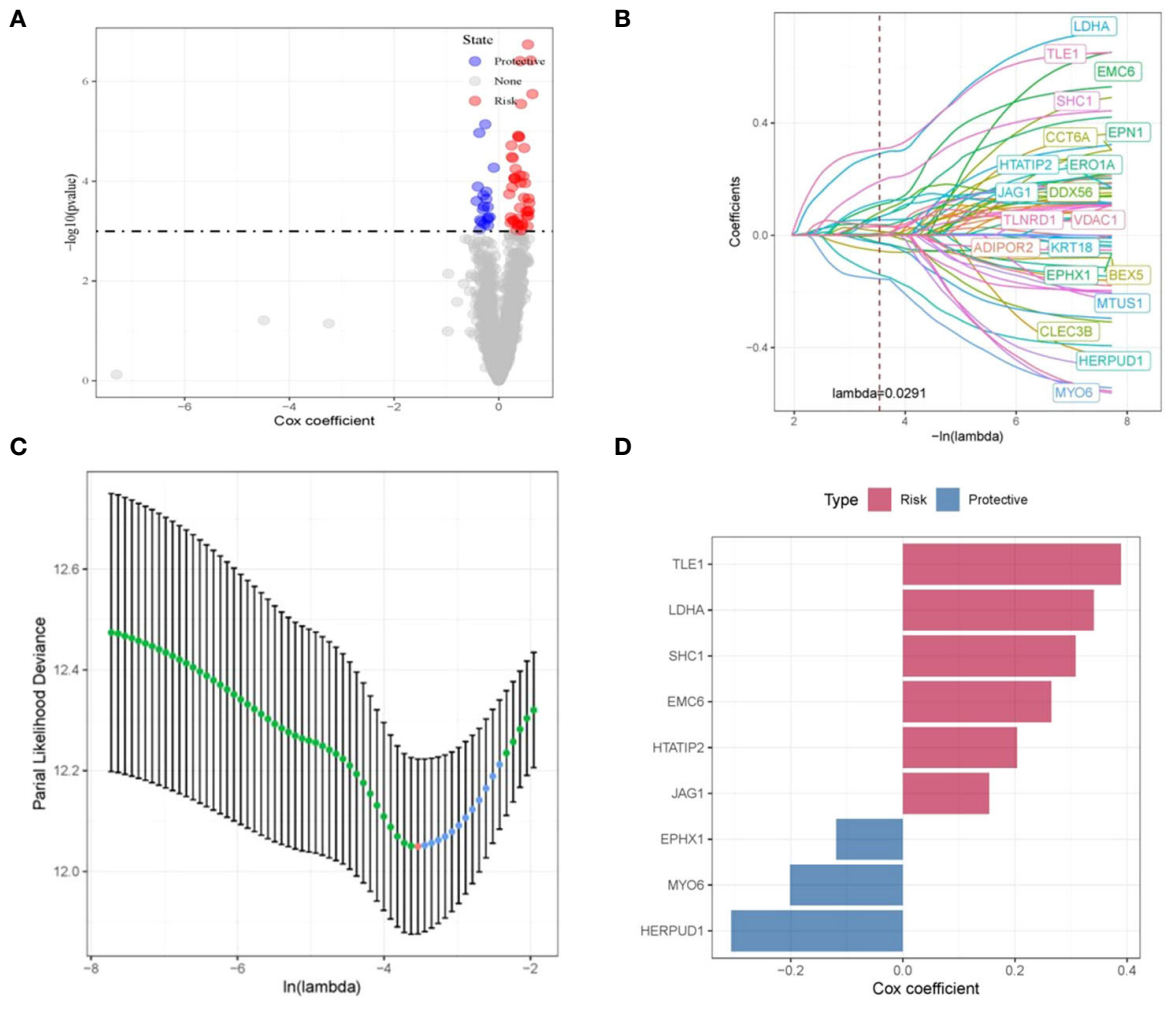
Construction and validation of disulfidptosis-related prognostic model. **(A)** Volcano plot illustrating the results of univariate Cox regression analysis for the intersecting genes. **(B, C)** Nine disulfidptosis-related genes exhibiting the strongest associations with survival status, identified via LASSO-Cox regression. **(D)** LASSO-Cox regression coefficients representing the nine identified genes.

The interplay between tumor cells and ECs results in vasculature formation, a pivotal factor in tumor initiation and advancement. Moreover, EC-driven metabolic pathways effectively meet the escalated energy demands of tumors, thus facilitating swift tumor proliferation. (20). Consequently, for a deeper exploration of disulfidptosis, we focused on ECs. We initiated our investigation by conducting a differential analysis of endothelial cell subpopulations between tumor and normal tissues, employing GSE149655, which yielded 1713 DEGs. To assess the potential impact of these disulfidptosis-related genes on the survival outcomes of LUAD patients, we leveraged the TCGA-LUAD dataset to execute univariate Cox regression analysis on the intersecting genes. As demonstrated in **Figure 3A**, a noteworthy correlation was identified between 256 disulfidptosis-related genes and Overall Survival (OS) in LUAD patients.

Subsequently, through the application of LASSO-Cox regression, we successfully identified a cluster of nine genes that collectively constitute a disulfidptosis-associated prognostic signature (DRPS). These genes consist of TLE1, LDHA, SHC1, EMC6, HTATIP2, JAG1, EPHX1, MYO6, and HERPUD1, as visually depicted in **Figure 3B**. The corresponding LASSO-Cox regression coefficients affiliated with these nine genes are visually summarized in **Figure 3C**. Within this gene ensemble, TLE1, LDHA, SHC1, EMC6, HTATIP2, and JAG1 were discerned as unfavorable prognostic biomarkers (cox coefficient > 1), whereas EPHX1, MYO6, and HERPUD1 manifested as favorable prognostic biomarkers (cox coefficient < 1), as illustrated in **Figure 3D**.

**Figure 4:**
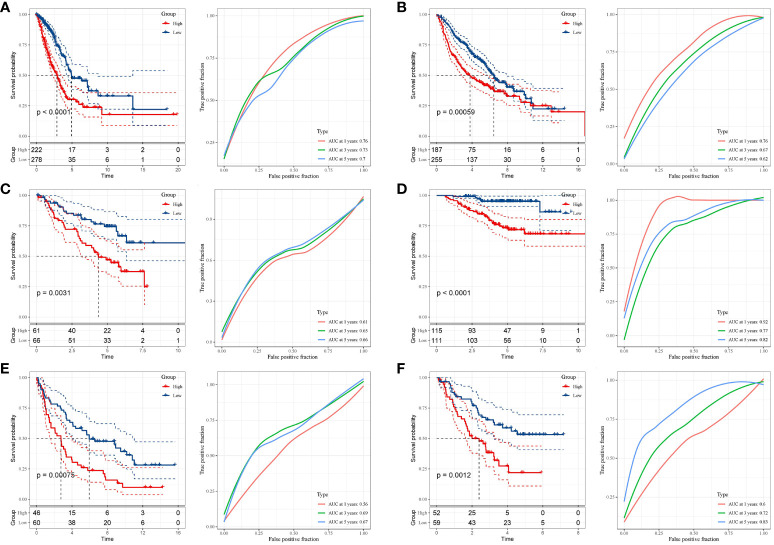
Validation of the risk signature. **(A–F)** Kaplan-Meier analysis demonstrating the Overall Survival (OS) disparity between high- and low-risk groups across the TCGA-LUAD, GSE68465, GSE50081, GSE37745, GSE31210, and GSE3141 datasets. Corresponding datasets were utilized for ROC analysis to gauge predictive efficiency.

Next, the risk score for every cancer sample was computed utilizing the subsequent equation: riskscore=(0.386×TLE1exp.)+ (0.321 × LDHA exp.)+ (0.309 × SHC1 exp.)+ (0.253× EMC6 exp.)+ (0.213 × HTATIP2exp.)+ (0.752 ×JAG1 exp.)+(-0.121 × EPHX1 exp.)+ (-0.203× MYO6 exp.)+ (-0.304 × HERPUD1 exp.). Based on this equation, it becomes feasible to compute an individual risk score for each patient. Subsequently, patients within distinct cohorts can be segregated into high- and low-risk categories based on the median value. Kaplan-Meier survival curves eloquently depicted that individuals situated in the high-risk cohort exhibited notably inferior OS outcomes compared to their counterparts in the low-risk cohort (p < 0.001). (**Figure 4A**). We evaluated the predictive capacity of our developed features using a time-dependent ROC analysis. The AUC values for 1-, 3-, and 5-year survival rates were 0.76, 0.73, and 0.70, respectively (**Figure 4A**). Additionally, we assessed the stability of the prognostic risk features across five internal validation sets: GSE68465, GSE50081, GSE37745, GSE31210, and GSE3141. The outcomes revealed a substantial discrepancy in Overall Survival (OS) between patients categorized as high-risk and those designated as low-risk (p=0.00059, p=0.0031, p=0.00075, p<0.0001, and p=0.0012) (**Figures 4B–F**). The AUC for the GSE68465 risk profile stood at 0.76 for 1 year, 0.67 for 3 years, and 0.62 for 5 years (**Figure 4B**). Correspondingly, the AUC for the GSE50081 risk profile was 0.61 for 1 year, 0.65 for 3 years, and 0.66 for 5 years (**Figure 4C**). The GSE37745 risk profile exhibited an AUC of 0.56 for 1 year, 0.69 for 3 years, and 0.67 for 5 years (**Figure 4D**). On the other hand, the GSE31210 risk profile demonstrated an impressive AUC of 0.92 for 1 year, 0.77 for 3 years, and 0.82 for 5 years (**Figure 4E**). Similarly, the GSE3141 risk profile showcased an AUC of 0.60 for 1 year, 0.72 for 3 years, and 0.83 for 5 years (**Figure 4F**). Notably, these internal validation sets consistently exhibited commendable AUC performance (**Figures 4B–F**).

### 3.4 Independent analysis of prognostic and clinicopathological features

**Figure 5:**
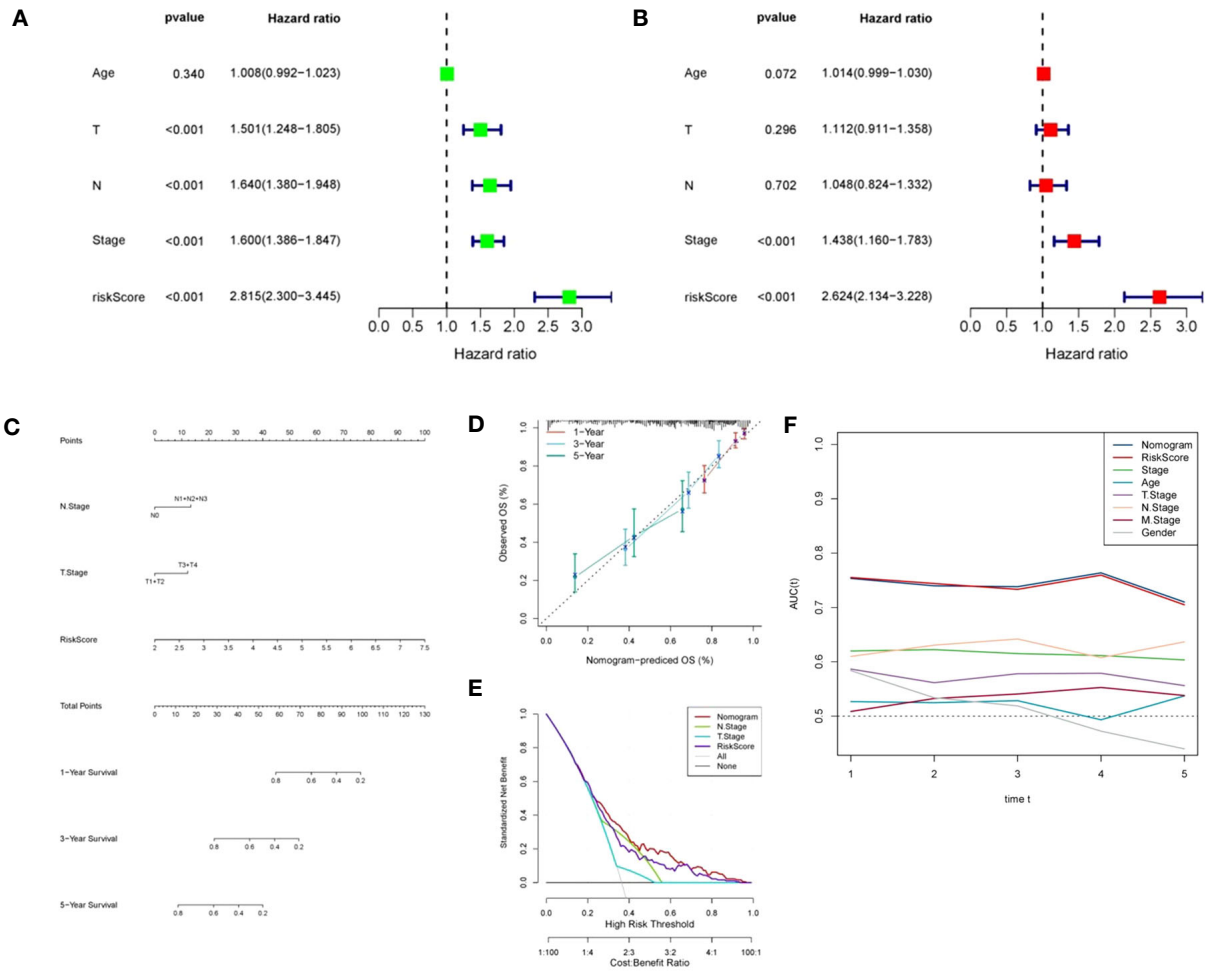
Independent prognostic analysis of DRPS. **(A, B)** Univariate and multivariate Cox analyses were conducted to assess the independence of risk score and clinicopathological attributes. **(C)** Construction of nomograms integrating risk scores and clinical variables to predict 1-, 2-, and 5-year survival. **(D)** Calibration curves gauging the alignment between observed outcomes and the predicted outcomes at 1-, 2-, and 5-year intervals. **(E)** Decision curve plot showcasing normalized net benefit: the y-axis represents standardized net benefit, while the x-axis delineates the threshold probability spectrum. **(F)** AUC values for nomogram, risk score, and each clinical feature.

**Figure 6:**
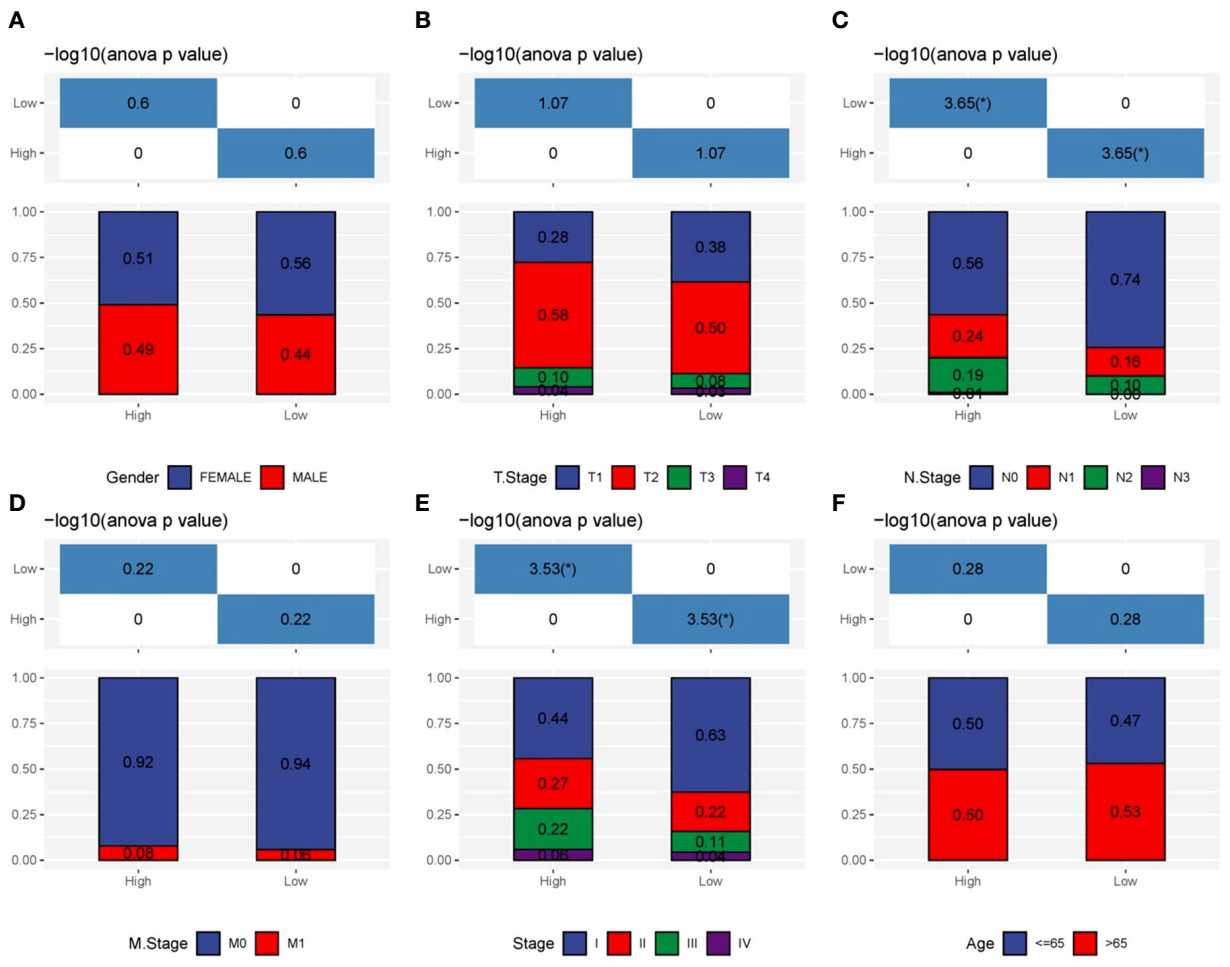
Clinicopathologic characteristics analysis. **(A–F)** The interrelations between risk signature and clinicopathologic parameters, encompassing gender, T stage, N stage, M stage, tumor stage, and age.

We assessed the predictive significance of DRPS and various clinical factors using both univariate and multivariate Cox regression analysis. The prognosis of LUAD was significantly associated with tumor stage, T stage, N stage, and risk score according to the results of univariate Cox regression analysis (**Figure 5A**). The risk score remained an independent prognostic factor in LUAD patients, as demonstrated by multivariate Cox regression analysis (**Figure 5B**). Using clinical data from the TCGA-LUAD dataset, we created a nomogram that displays scores and clinical variables based on DRPS (**Figure 5C**). The calibration curves further revealed satisfactory concordance between observed and projected survival rates at 1-, 3-, and 5-year intervals. (**Figure 5D**). For a more comprehensive assessment of the nomogram’s effectiveness, we juxtaposed it with other prognostic variables. The decision curve analysis demonstrated that our nomogram yielded the most favorable net benefit in comparison to the clinical factors. (**Figure 5E**). Furthermore, ROC curves illustrated that the Area Under the Curve (AUC) of both the nomogram and risk score outperformed other clinical attributes, reaching the highest values (**Figure 5F**). After a more in-depth investigation and examination of the clinicopathological features of LUAD patients in the TCGA dataset, the differences between the high-risk score group and the low-risk score group in terms of gender, T stage, N stage, M stage, tumor stage, and age were as follows: There were no significant disparities in gender, T stage, M stage, and age (**Figures 6A, B, D, F**), but there were notable differences in the N stage and tumor stage (**Figures 6C, E**).

### 3.5 Functional analysis

**Figure 7:**
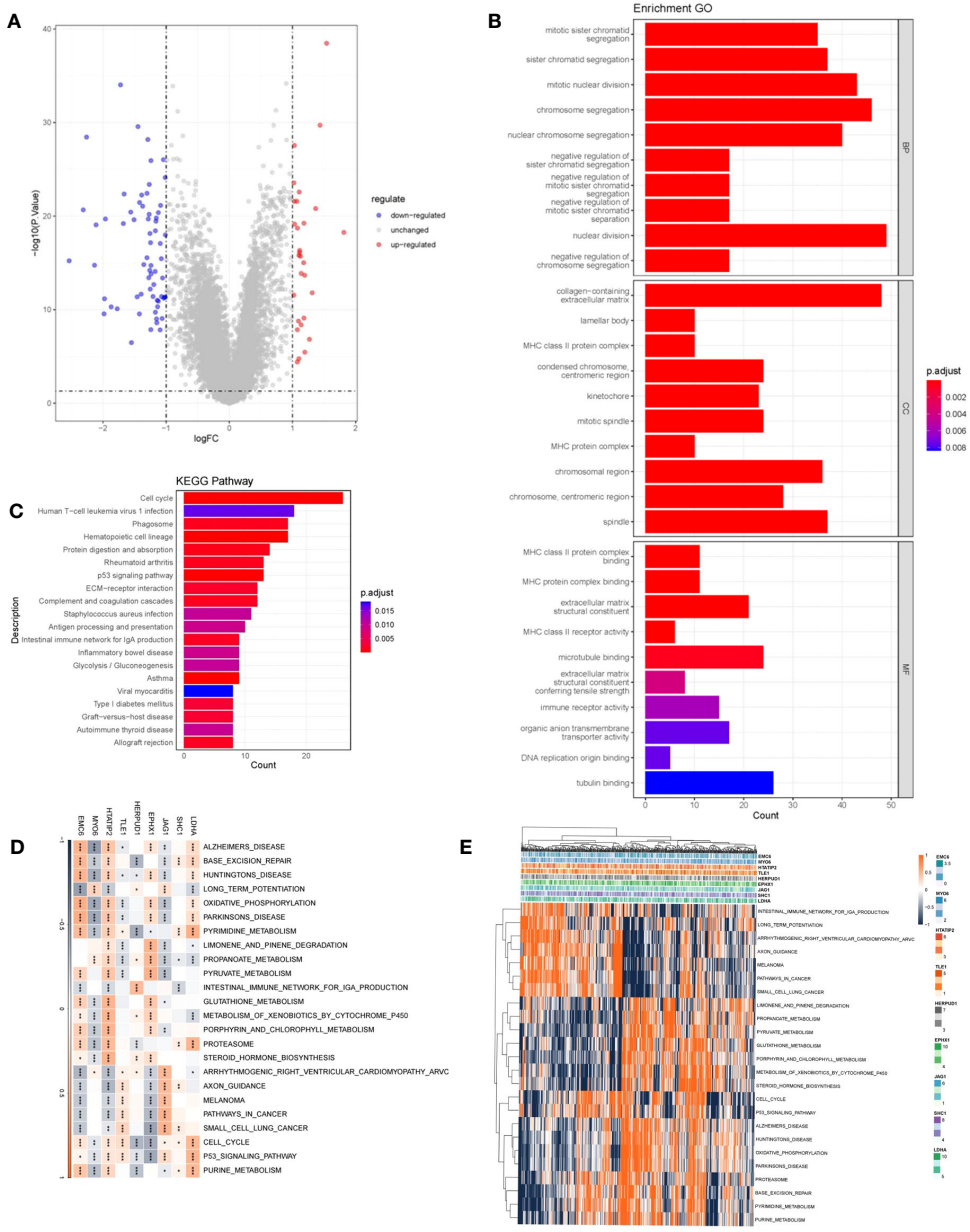
Functional analysis. **(A)** Volcano plot depicting DEGs between high- and low-risk groups. Red indicates up-regulated genes, blue indicates down-regulated genes, and gray indicates genes with no significant difference. **(B)** Histogram presenting the outcomes of GO enrichment analysis. **(C)** Histogram presenting the results of KEGG-based enrichment analysis. **(D)** Heatmap illustrating the relationship between prognostic genes and hallmark pathways (*p < 0.05; **p < 0.01; ***p < 0.001). **(E)** Heatmap displaying the expression levels of prognostic genes within hallmark pathways.

To explore DRPS-related biological functions and pathways, we detected DEGs between high- and low-risk groups, resulting in 610 DEGs (**Figure 7A**). Subsequently, we performed GO and KEGG enrichment analysis on these DEGs. The analysis revealed significant enrichment in cell cycle-related pathways like nuclear division and chromosome segregation (**Figures 7B, C**). Additionally, we conducted GSVA enrichment analysis for the nine prognostic genes. The analysis indicated strong associations of these genes with signaling pathways including huntington’s disease, propanoate metabolism, oxidative phosphorylation, metabolism, cell cycle, and the p53 signaling pathway (**Figure 7D**). Expression patterns of these prognostic genes within these pathways were depicted using a heatmap (**Figure 7E**).

### 3.6 Mutation frequency and instability measures of prognostic genes

**Figure 8:**
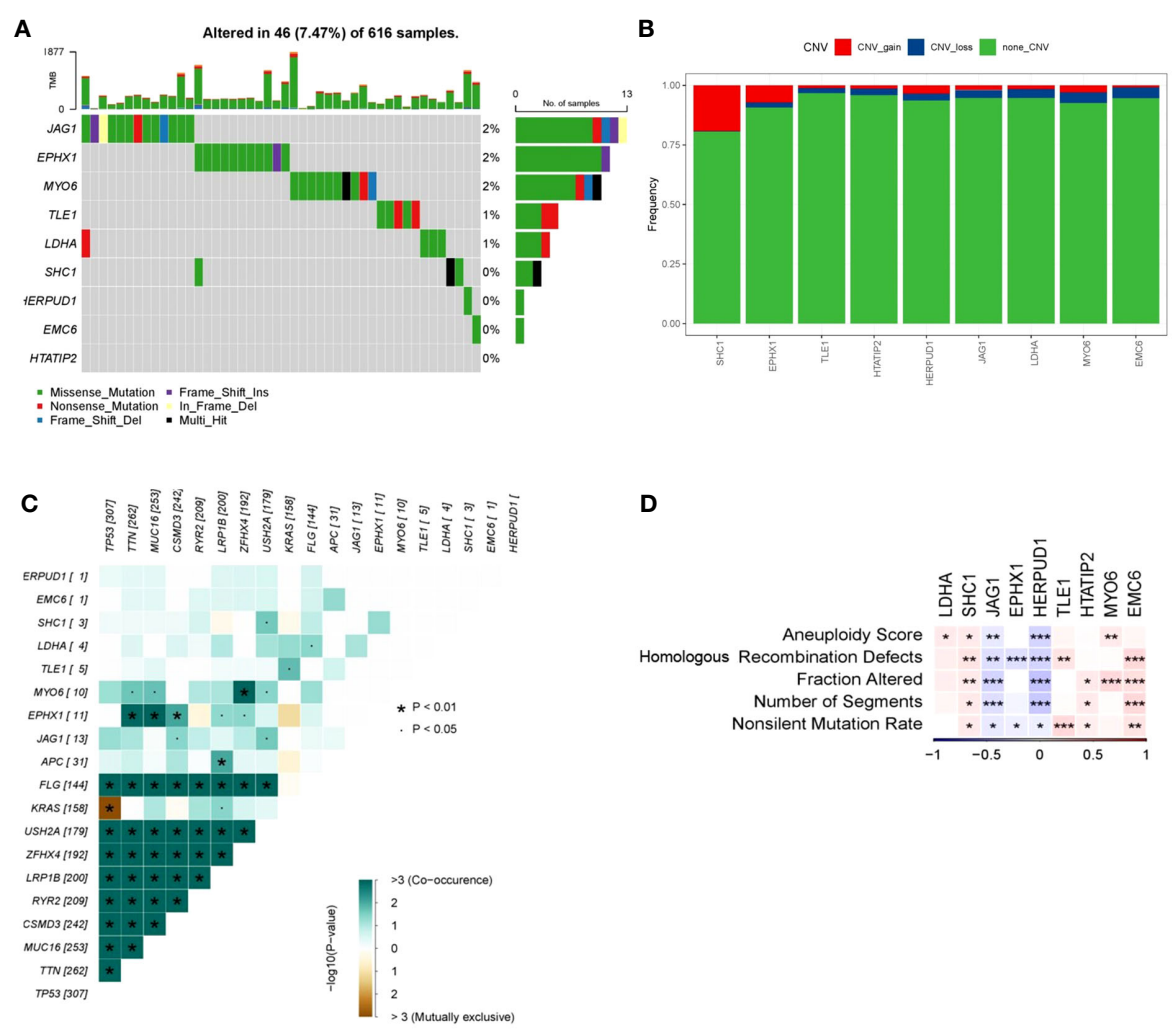
Mutation frequency and instability measures of prognostic genes. **(A)** Waterfall plot illustrating mutations within prognostic genes. **(B)** Heatmap showcasing co-occurrence and mutually exclusive mutations among differentially mutated genes (*p < 0.01 and p < 0.05). **(C)** Frequencies of CNV gain, loss, and non-CNV genes derived from LASSO regression. **(D)** Associations between DNA damage metrics and prognostic genes across various genes. Heatmap presenting instability measures of prognostic genes for TCGA-LUAD (*p < 0.05, **p < 0.01, and ***p < 0.001).

To gain deeper insights into the mechanistic underpinnings through which the DRPS risk score signature effectively gauges patient prognosis, we delved into the mutational landscape of the nine prognostic genes. Employing the Maftools R package, we scrutinized the mutation status of these genes, and the analysis revealed several intriguing findings (**Figure 8A**). Among the identified mutations, we observed that the five most commonly mutated genes were JAG1, EPHX1, MYO6, TLE1, and LDHA. These mutations highlight the potential relevance of these genes in the context of LUAD. Additionally, we investigated Copy Number Variation (CNV) events within the nine prognostic genes and found that SHC1 exhibited significant CNV gain, while MYO6 and EMC6 displayed noteworthy CNV loss (**Figure 8B**).Upon assessing the co-occurrence and mutually exclusive interactions among the top 19 mutated genes within the high- and low-risk groups, we observed a prevalent pattern of gene mutation co-occurrence involving numerous genes. Notably, the most prominent of these interactions was the mutual exclusion between TP53 and KRAS mutations, as prominently depicted in **Figure 8C**. This observation could have important implications for understanding tumor biology and treatment response in LUAD. The recurrent mutations in the TP53 gene, which serve as a hallmark of LUAD and signify elevated genome instability, were also noteworthy. These mutations may contribute to tumor development and progression, potentially affecting patient prognosis and treatment outcomes. (6, 21). Taking into account the fact that increased tumor mutational burden in hereditary LUAD isn’t linked with heightened immune activity in the tumor microenvironment, our exploration extended to other indicators of instability that could impact the immune response in LUAD. Utilizing iAtlas, we delved into various markers of genomic instability within LUAD. Interestingly, our findings unveiled a positive correlation between LDHA, SHC1, and MYO6 with aneuploidy score, while JAG1 and HERPUD1 displayed a negative correlation (**Figure 8D**). SHC1, TLE1, and EMC6 displayed positive correlations with homologous recombination defects and nonsilent mutation rate, whereas JAG1, EPHX1, and HERPUD1 exhibited significant negative correlations with these factors (**Figure 8D**). Furthermore, in terms of fraction altered, SHC1, HTATIP2, MYO6, and EMC6 demonstrated positive correlations, while JAG1 and EPHX1 displayed significant negative correlations (**Figure 8D**). Similarly, SHC1, HTATIP2, and EMC6 exhibited positive correlations with the number of segments, whereas JAG1 and EPHX1 displayed significant negative correlations (**Figure 8D**).

These findings collectively suggest a link between genomic instability and patients’ survival risk scores. However, the relationship between measures of genomic instability and tumor immune activity is intricate and necessitates further exploration.

### 3.7 Immune landscape analysis

**Figure 9:**
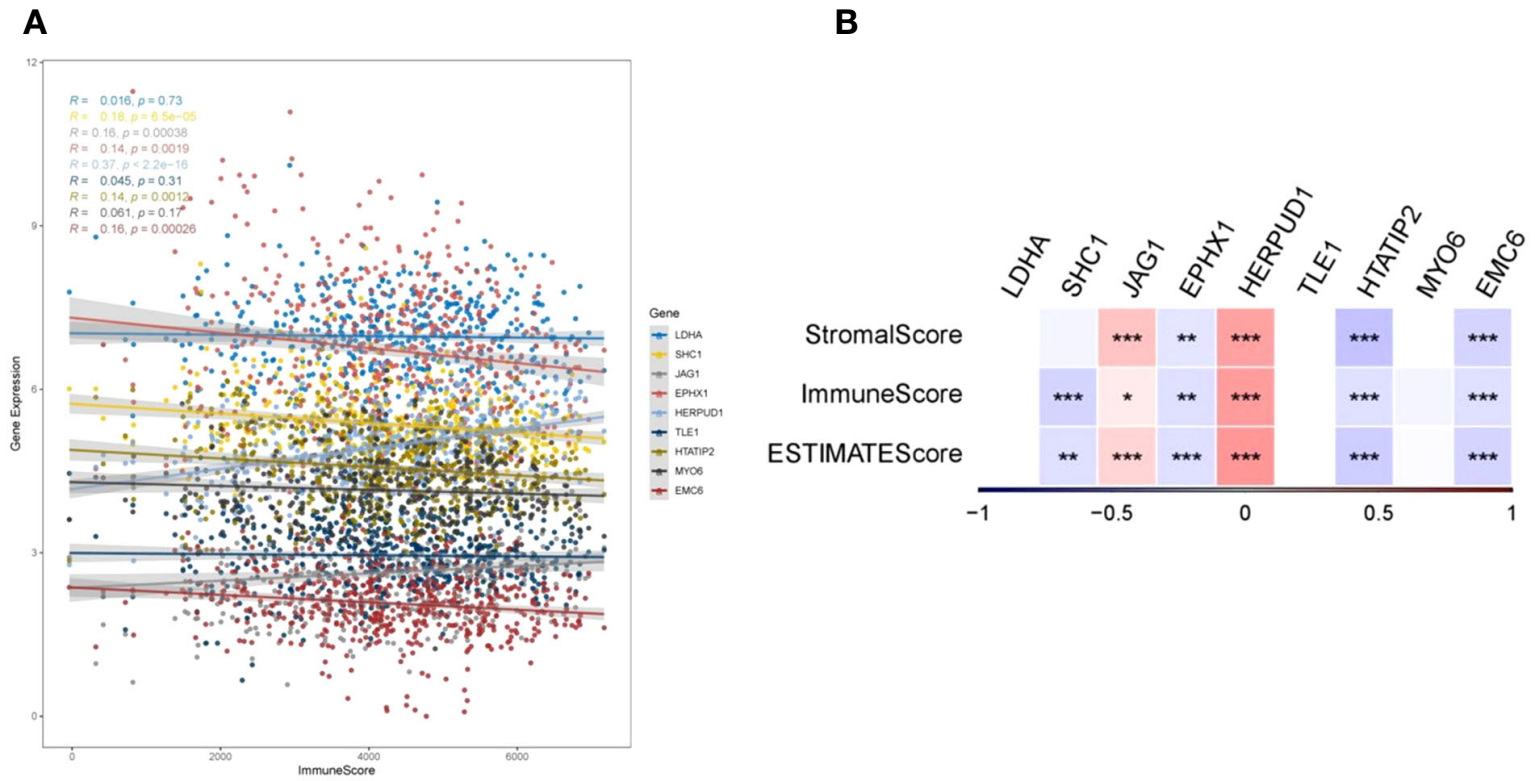
TumorPurity analysis. **(A)** Correlations between gene expression levels and ImmuneScore. **(B)** Correlations of the nine prognostic genes with ImmuneScore, StromalScore, and ESTIMATEScore (*p < 0.05, **p < 0.01, and ***p < 0.001).

**Figure 10:**
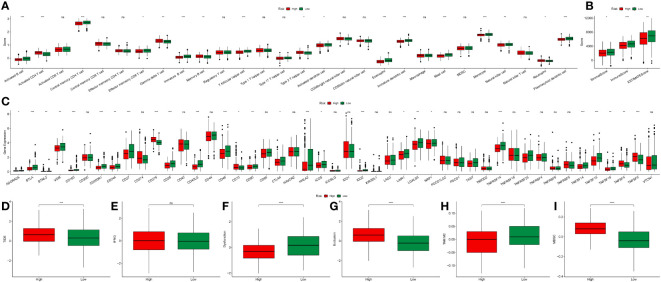
Immune landscape analysis. **(A)** Boxplot illustrating scores of 28 immune cell types between high- and low-risk groups. **(B)** Boxplot demonstrating ImmuneScore, StromalScore, and ESTIMATEScore differences between high- and low-risk groups. **(C)** Boxplot depicting the expression variation of immune-related genes between high- and low-risk groups. **(D–I)** Boxplots illustrating differences in TIDE, IFNG, T cell dysfunction score, T cell exclusion score, TAM M2, and MDSC cell proportions between high- and low-risk groups (*p < 0.05, **p < 0.01, and ***p < 0.001).

To elucidate the intricate relationship between DRPS and the tumor microenvironment, we leveraged the ESTIMATE algorithm to assess the influence of the nine prognostic genes. The outcomes underscored the positive correlation of JAG1 and HERPUD1 with StromalScore, ImmuneScore, and ESTIMATEScore. Conversely, EPHX1, HTATIP2, and EMC6 demonstrated negative correlations with these scores (**Figures 9A, B**). Furthermore, SHC1 exhibited a notable negative correlation with ImmuneScore and ESTIMATEScore (**Figure 9B**). Intriguingly, our investigation revealed that individuals categorized in the low-risk group exhibited greater tumor purity in comparison to those in the high-risk group. Moreover, the proportions of immune cells and stromal cells within tumors of low-risk group patients surpassed those in the high-risk group (**Figure 10B**). Given the pivotal role of immune score in prognosis assessment, we conducted an in-depth investigation to ascertain whether the survival discrepancy between high-risk and low-risk patients could be attributed to the immune microenvironment within tumors. Consequently, we scrutinized the infiltration of 28 distinct immune cell types across the immune microenvironment in tumors from both high- and low-risk patient groups. The findings unveiled that compared to the low-risk group, individuals in the high-risk group exhibited diminished counts of activated B cells, NK cells, eosinophils, mast cells, monocytes, and plasmacytoid dendritic cells within their tumors. Conversely, the high-risk group displayed heightened presence of cells like activated CD4 T cells, dendritic cells, and neutrophils (**Figure 10A**). The majority of functional immune cell infiltrations were observed within the low-risk group (**Figure 10A**), indicative of an association between the risk score and the immune microenvironment. Additionally, our findings indicated that in the high-risk group, the expression of immune suppression-related genes such as CD276, CD70, CD80, and TNFSF9 was significantly elevated compared to the low-risk group. Conversely, the high-risk group displayed decreased expression of immune activation-related genes like CD28, CD48, TNFSF14, and others (**Figure 10C**). This holds promise for predicting potentially responsive drugs in high-risk patients. The tumor immune response stands pivotal in immunotherapy. Given the positive correlation between the TIDE predictive score and tumor immune evasion, the TIDE prediction score emerges as an effective tool for evaluating tumor immune escape within high- and low-risk groups. In the TCGA-LUAD dataset, we observed that the TIDE score was notably higher in the high-risk score group than in the low-risk score group (**Figure 10D**). Meanwhile, aside from Interferon gamma (IFNG) which showed no significant difference between the two groups (**Figure 10E**), there were distinct disparities between the groups in terms of T cell dysfunction score, T cell exclusion score, TAM M2, and MDSC cell proportions (**Figures 10F–I**). These collective findings suggest that patients with a high-risk score may have limited benefits from immunotherapy.

### 3.8 Predictive value of DRPS in immunotherapy

**Figure 11:**
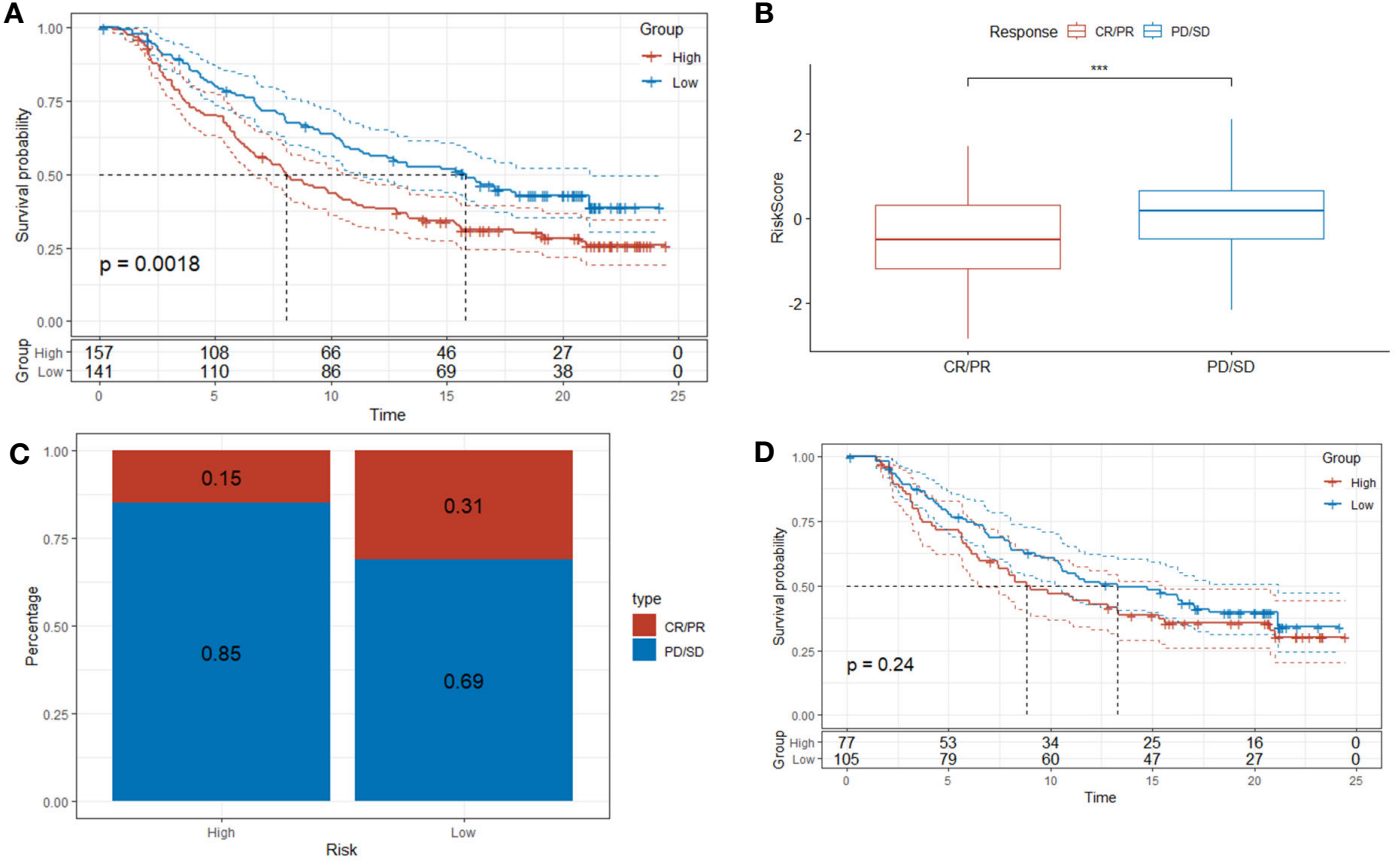
Predictive value of DRPS in immunotherapy. **(A–C)** Investigating the efficacy of DRPS as a predictive indicator for immunotherapy using the Imvigor210 dataset. **(D)** Based on the IMvigor210 dataset, Kaplan-Meier analysis was performed on the risk scores of clinical stages I and stages II patients. (***p < 0.001).

To bolster the robustness of DRPS’s predictive potential, we subjected it to external validation using the IMvigor210 dataset to assess patients’ responsiveness to immunotherapy. This validation aligns with prior findings, as the Kaplan-Meier analysis once again affirmed that individuals in the high-risk group faced less favorable overall survival (OS) in contrast to those in the low-risk group (**Figure 11A**). Furthermore, our investigation unveiled that patients who exhibited positive responses to immunotherapy (CR/PR) demonstrated notably lower risk scores in comparison to those who did not respond as favorably (SD/PD) (**Figure 11B**). As illustrated in **Figure 11C**, the response rate to immunotherapy was significantly higher within the low-risk group than in the high-risk group (31% versus 15%, respectively). In addition, for a more comprehensive assessment of DRPS's predictive capabilities, we conducted Kaplan-Meier analysis on patients with clinical stages I-II (**Figure 11D**). This not only further reinforces the resilience of DRPS but also underscores its substantial predictive utility in guiding clinical treatment decisions.

### 3.9 Pseudotime analysis

**Figure 12:**
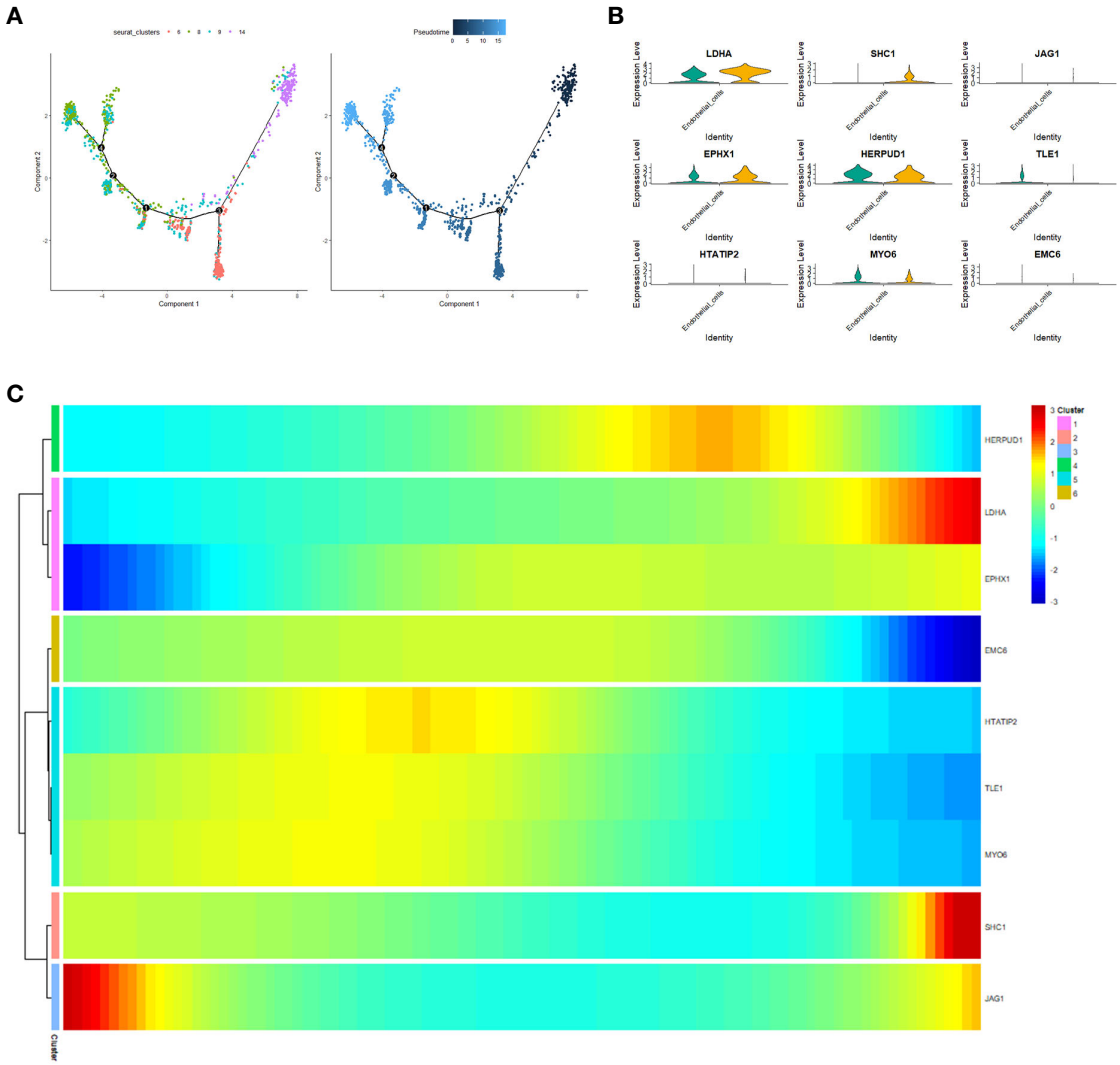
Pseudotime analysis. **(A)** Cellular trajectories for fourteen distinct endothelial cell subsets. Each dot corresponds to a cell, while the black line denotes the slingshot trajectory. **(B)** Heatmap visualizing the expression of the nine prognostic genes (log-normalized counts, represented by color) categorized by respective pseudotimes and cell clusters. **(C)** Violin plot illustrating the expression variance of the nine prognostic genes in endothelial cells (ECs) between tumor tissue and normal tissue.

To unravel the malignant evolution pattern of endothelial cells (ECs) within LUAD, we harnessed the Monocle R package for cell trajectory analysis. Illustrated in **Figure 12A** are the cell trajectories for fourteen distinct endothelial cell clusters, and the trajectory orientation dictated by unsupervised pseudotime. This arrangement facilitated the identification of branch points, multiple branches, and nodes that permeate developmental trajectories, where cells within the same branch reflected a shared state (**Figure 12A**). This progressive process depicts the transformation of normal ECs into characteristic malignant ECs characteristic of LUAD. Concurrently, alterations in the expression levels of the nine prognostic genes transpired over pseudotime across various cell clusters. Specifically, along the trajectory of pseudotime progression, LDHA, EPHX1, and SHC1 exhibited gradual increases in expression, while EMC6, HTATIP2, TLE1, MYO6, and JAG1 demonstrated diminishing expressions (**Figure 12B**). Expanding our analysis to a single-cell RNA-seq dataset (GSE149655), we juxtaposed the expression disparities of the nine prognostic genes in ECs between tumor tissues and normal tissues. Notably, LDHA, SHC1, and EPHX1 exhibited heightened expression levels in tumor tissues, while MYO6 and TLE1 displayed diminished expression compared to normal tissues (**Figure 12C**).

### 3.10 Drug prediction and qRT-PCR validation

**Figure 13:**
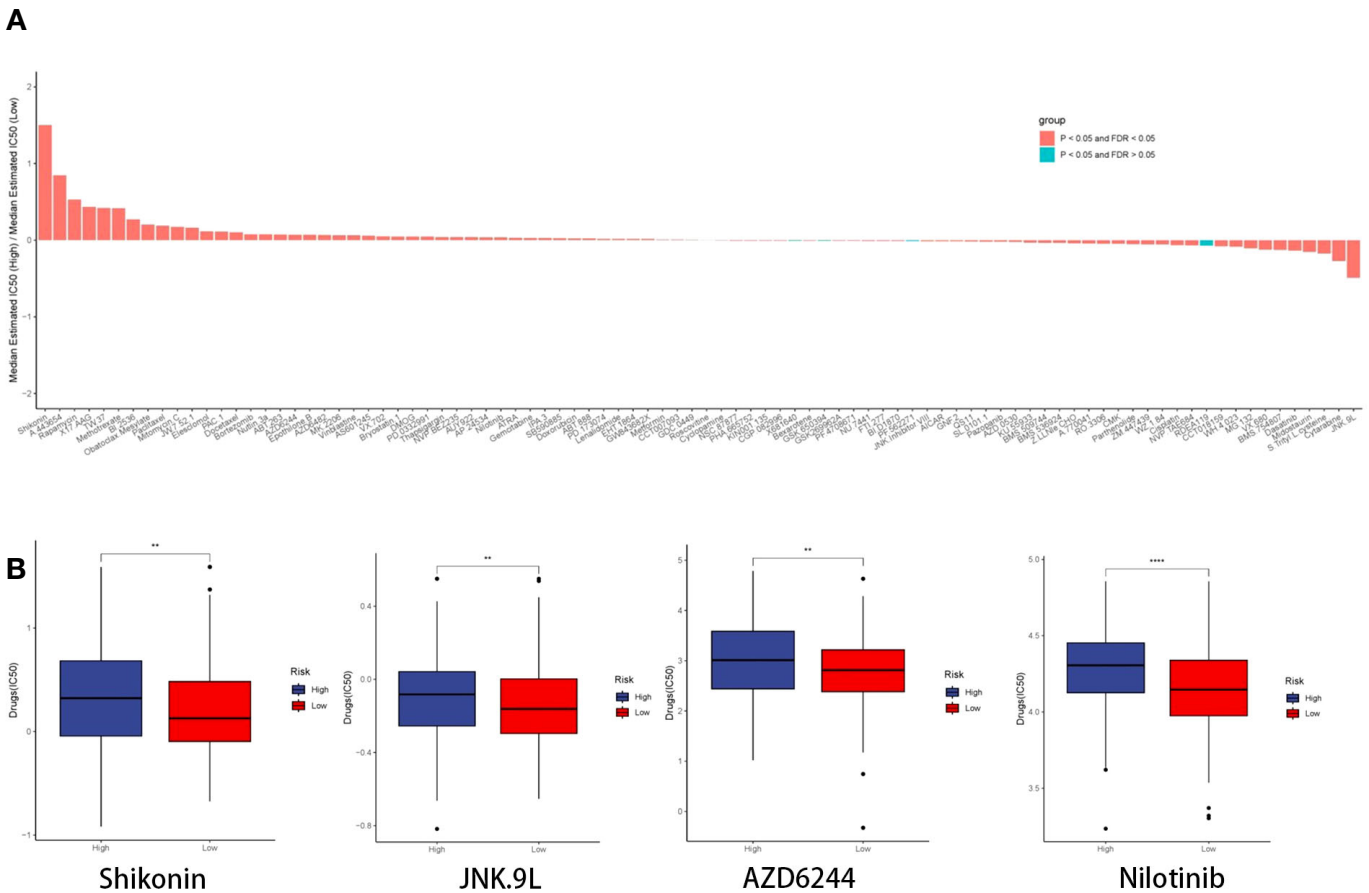
Drug prediction and qRT-PCR validation. **(A)** Histogram depicting the ratio of the median estimated IC50 of the high-risk group to that of the low-risk group for each compound. **(B)** Boxplot illustrating the IC50 profiles of the four compounds across the high- and low-risk groups.

**Figure 14:**
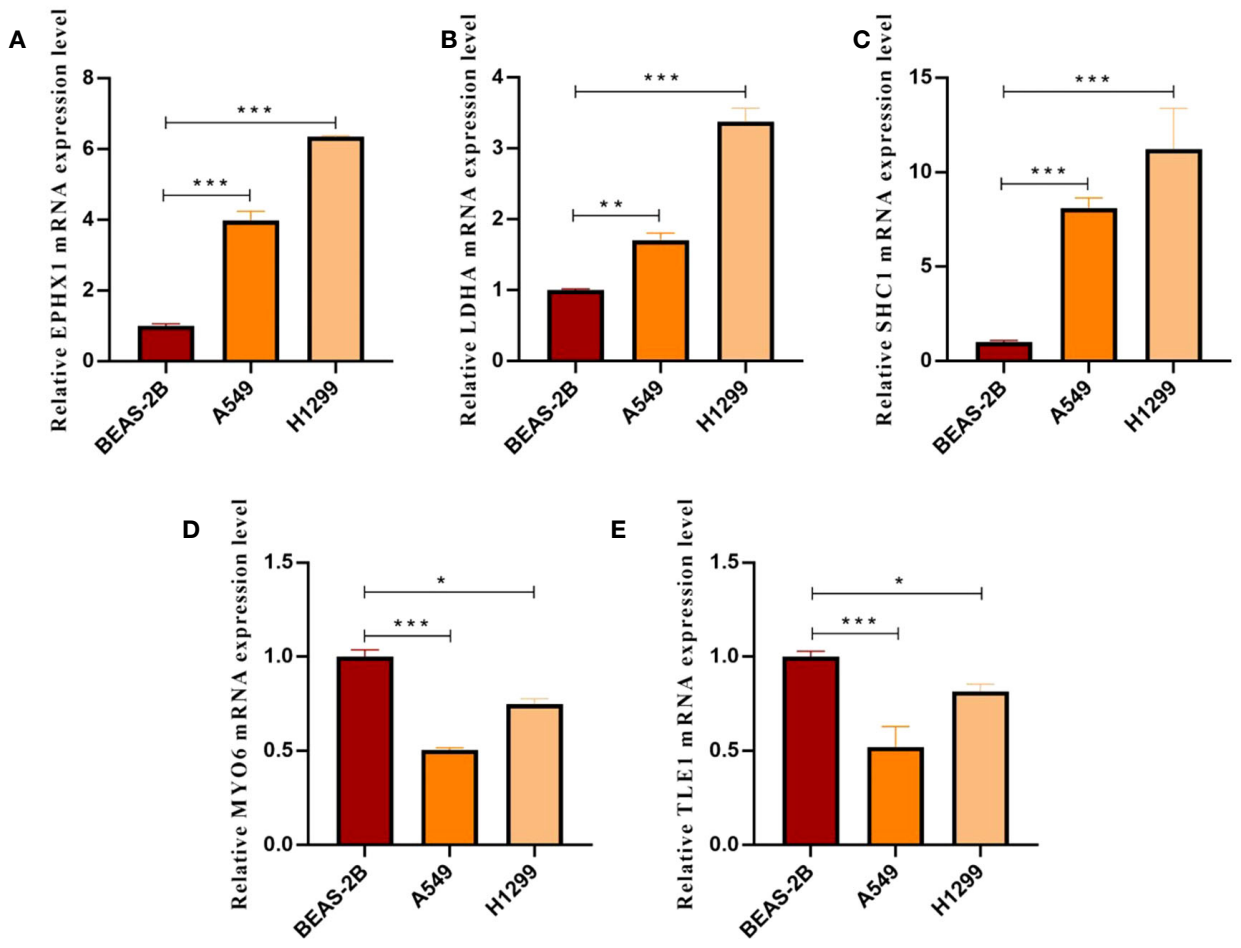
**(A–E)** Histograms presenting qRT-PCR outcomes for EPHX1, LDHA, SHC1, MYO6, and TLE1, respectively. (*p < 0.05, **p < 0.01 and ***p < 0.001).

We employed the CGP database and the pRRophetic R package to anticipate the clinical responses of high- and low-risk patients to a variety of compounds (**Figure 13A**). Applying the criteria of P < 0.05 and FDR > 0.05, we identified four compounds: Shikonin, JNK.9L, AZD6244, and Nilotinib, which emerged as sensitive drugs within the low-risk population (**Figure 13B**). To reinforce the validity of our analysis, we assessed the expression levels of the nine prognostic genes in human non-small cell lung cancer cells (A549 and H1299) as well as human normal lung epithelial cells (BEAS-2B). The qRT-PCR results unveiled heightened expressions of EPHX1, LDHA, and SHC1 in A549 and H1299, whereas MYO6 and TLE1 exhibited lower expressions in A549 and H1299 when compared to BEAS-2B (**Figures 14A–E**). This outcome concurs with the trends identified in our prior analyses.

### 3.11 Validation and impact of LDHA knockdown on cellular proliferation

**Figure 15:**
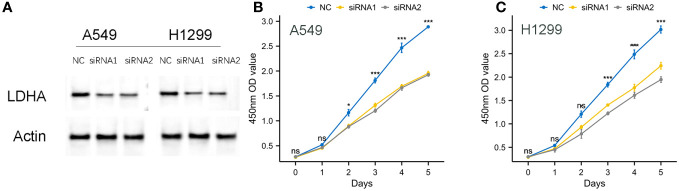
Knockdown of LDHA protein on cell proliferation viability. **(A)** Effective Knockdown of LDHA Protein in A549, H1299 Cells via siRNA. **(B, C)** Inhibition of Cellular Proliferative Vitality in LDNA Knockdown Group by CCK8 Assay.

To further verify the validity of our model, we designed small interfering RNA for LDHA and ascertained its ability to effectively knock down the target protein in A549, H1299 cells, (**Figure 15A**). Subsequently, a CCK8 assay was conducted, and the results of the experiment showed that the cellular proliferative vitality in the LDNA knockdown group was significantly inhibited, (**Figures 15B, C**).

## 4 Discussion

Lung adenocarcinoma (LUAD) stands as one of the most widespread malignancies across the globe. The clinical implementation of targeted therapy and immunotherapy has led to noteworthy enhancements in the prognosis of individuals with advanced stages of LUAD (6). Nevertheless, challenges persist in the realm of LUAD diagnosis and treatment, including issues such as delayed diagnosis, drug resistance, adverse events, and instances of non-response. These challenges continue to present significant hurdles in the clinical management of LUAD. (22). Hence, the identification and utilization of precise and responsive indicators are vital for the detection and management of LUAD. Since the complexity and heterogeneity of tumor tissue, bulk RNAseq dataset alone cannot infer the interaction and specific mechanism between cells in the tissue. By utilizing scRNA-seq dataset, correlative investigations can offer fresh perspectives on the progression of cancer, the diversity within tumors, and the microenvironment surrounding the tumor. Furthermore, as LUAD is a solid neoplasm, angiogenesis plays a crucial part in its progression, infiltration, and spread. Endothelial cells (ECs), acting as stromal cells within solid tumors, have a crucial function in the regulation of angiogenesis (23). Controlling endothelial cells to impact angiogenesis is a primary area of focus in cancer research. Disulfidptosis, a novel form of RCD, not only connects cellular metabolism to cellular destiny but also has a significant impact on the immune response to tumors (7, 8). Current studies related to disulfidptosis have suggested its great value in LUAD.

This research combined single-cell RNA sequencing and bulk RNA sequencing to provide a more comprehensive understanding of the involvement of disulfidptosis in LUAD. Additionally, a DRPS was developed for LUAD patients by identifying disulfidptosis-related genes that were differentially expressed in ECs. Our discovery revealed that the utilization of DRPS successfully categorized individuals into high-risk and low-risk clusters. In addition, our findings indicated that increased risk scores were linked to worse prognosis, decreased immune infiltration, and immunosuppressive condition. Hence, individuals with reduced risk scores might require immunotherapy to a greater extent.

This study suggests a prognostic gene panel called DRPS, which includes nine genes: TLE1, LDHA, SHC1, EMC6, HTATIP2, JAG1, EPHX1, MYO6, and HERPUD1. Following the analysis of bioinformatics data and subsequent *in vitro* experiments, we observed notable disparities in the levels of EPHX1, LDHA, SHC1, MYO6, and TLE1 expressions between LUAD and normal samples.

EPHX1 encodes the microsomal epoxide hydrolase 1 (MEH1), which has a twofold function in the reaction to carcinogenic substances found in the environment. EPHX1 not only metabolizes environmental carcinogens into trans-dihydrodiols, which can have mutagenic, toxic, and carcinogenic effects, but also generates substances necessary for detoxification processes (24–26). According to reports, the presence of EPHX1 gene polymorphisms has been linked to the susceptibility of different types of cancers (26, 27). The low variety of EPHX1 genotypes may reduce the risk of lung cancer (25). As one of the key enzymes of glycolysis, LDHA (lactate dehydrogenase A) will preferentially convert pyruvate into lactate under anaerobic conditions (28, 29). LDHA (lactate dehydrogenase A), a crucial enzyme in glycolysis, will primarily transform pyruvate into lactate. Cancer cells possess significant metabolic flexibility, enabling them to choose substrates depending on their accessibility. Tumor cells situated in areas with low oxygen levels heavily rely on glucose-fueled anaerobic glycolysis, leading to the oxidation of glucose into pyruvate or lactate (29). O Metabolic reprogramming in response to hypoxic stress, which is crucial for meeting proliferative demands, is considered a key characteristic of malignancy (30). Thus, LDHA can guarantee the metabolic flexibility of cancer cells, allowing them to adjust to challenging conditions (31). At the same time, LDHA not only provides energy for T cells, but also the acidic microenvironment formed with lactic acid can also function as a barrier for T cells (32, 33). Modulation of LDHA has considerable potential value for T cell activation-based immunotherapy. According to reports, LDHA has the ability to enhance the advancement of LUAD by controlling molecules associated with epithelial-mesenchymal transition (EMT) (34). Certain LDHA inhibitors at the molecular level have notable impacts on tumor load, metastasis, and cellular demise (32). Nonetheless, only a limited number of studies have assessed alterations in immune cell reactions towards LDHA inhibitors during cancer treatment (32). Therefore, targeting LDHA can create new opportunities to fight cancer cells (31). SHC1 (SHC adapter protein 1) integrates and transduces external stimuli into distinct signaling networks (35, 36). Recent research has indicated that the SHC1 gene has a significant impact on different types of tumors, including breast cancer and gastric cancer (37, 38). Bioinformatics analysis revealed that elevated SHC1 expression served as a prognostic indicator for unfavorable outcomes in numerous cancer types (39–41). According to reports, SHC1 has the potential to control the signaling pathway of the epidermal growth factor receptor (EGFR), subsequently triggering multiple downstream signaling pathways like MAPK/ERK, PI3K/Akt, and STAT3. These pathways play a crucial role in facilitating the metastasis of lung cancer (42, 43). Nevertheless, additional investigation is required to explore the relationship between SHC1 and EGFR.As a unique member of the myosin superfamily, MYO6 (myosin VI) has a unique orientation, which helps it play a key role in endocytosis, vesicle trafficking, protein secretion and autophagosome maturation (44, 45). According to reports, the excessive expression of MYO6 has been linked to a malignant characteristic in individuals diagnosed with different types of cancer, such as prostate and gastric cancer (46, 47). The upregulation of MYO6 is found to be associated with maintaining the cell cycle and cell growth in lung cancer cells (48). Research has indicated that non-coding RNA (miRNA) has the ability to impact the growth and advancement of tumors by controlling the expression of MYO6.In NSCLC cells, miR-5195-3p functions as a cancer inhibitor through the direct regulation of MYO6 expression (49). Furthermore, TLE1 functions as a suppressor of numerous signaling pathways via transcription factors, exerts control over the transcriptional activity of diverse genes, and exhibits a range of physiological roles (50). Notably, research has discovered that TLE1 has the ability to facilitate the suppression of the E-cadherin gene, which is a crucial controller of EMT in lung cancer cells. As a result, this promotes the advancement of tumors (51). While the exact mechanism is yet to be investigated, these findings indicate that the aforementioned predictive genes have the ability to not just monitor individuals with LUAD, but also have the potential to be targeted for therapeutic purposes.

Furthermore, research indicates that EMC6, HTATIP2, HERPUD1, and JAG1 also have a significant impact on the formation and progression of tumors. Overexpression of EMC6, a subunit of the protein complex found in the endoplasmic reticulum membrane, has been shown to suppress cancer cell growth and trigger apoptosis. This gene is responsible for encoding autophagy-related proteins (52–55). Tumor metabolic reprogramming is regulated by HTATIP2, which functions as a tumor suppressor. According to reports, when tumor cells experience hypoglycemic conditions, the absence of HTATIP2 expression can enhance the cells’ metabolic adaptation to glucose limitation (56). The increased sensitivity of LUAD to EMT processes activated by drug therapy and the enhancement of tumor metabolic plasticity, regulation of tumor adaptation to hypoxia, and promotion of an aggressive tumor phenotype are consequences of the deficiency in HTATIP2 expression (57). HERPUD1, a crucial component of the degradation complex involved in ER-associated degradation (ERAD), has the ability to control ERAD (58). And not only that, HERPUD1 plays a role in protein degradation and stress (59). JAG1, a cell surface ligand of the Notch signaling pathway, is highly expressed in numerous cancers that are closely associated with tumor biology and is inversely associated with the prognosis of these cancers (60–62). The primary reason for this is because JAG1/Notch signaling governs malignant cellular processes and triggers numerous cancer-causing elements that oversee functions like spread of cancer, resistance to drugs, formation of new blood vessels, and properties resembling stem cells via signaling sequences (63, 64). Furthermore, there have been reports indicating that JAG1 might be indicative of resistance to immunotherapy in LUAD and is linked to a repressive immune microenvironment (65). Pseudotime analysis revealed changes in the expression of EMC6, HTATIP2, HERPUD1, and JAG1 in various cell clusters during the progression of LUAD, although no significant differences were observed in ECs between LUAD and normal samples. Monitoring the patient’s condition and adjusting the treatment plan can be done more effectively by detecting these markers.

In conclusion, the CGP database predicted Shikonin, Selumetinib, Nilotinib, and JNK.9L as potential drugs for the low-risk score group. The primary ingredient of Comfrey, known as Shikonin, acts as an inhibitor that effectively suppresses the key enzyme pyruvate kinase M2 in glycolysis. It possesses diverse effects including antioxidation, anti-inflammatory properties, and anti-tumor activity (66–68). Numerous studies indicate that shikonin has the ability to impact the invasion, proliferation, viability, and drug resistance of lung cancer (69–71). AZD6244 (Selumetinib) is a mitogen-activated protein kinase 1 and 2 (MEK1/2) inhibitor for the treatment of neurofibromatosis and various tumors (72). Selumetinib has been extensively evaluated in patients with NSCLC. Research conducted solely with this medication has not shown its effectiveness in treating NSCLC. In patients with chemotherapy-pretreated KRAS-mutant NSCLC, the response rates and progression-free survival were enhanced by the inclusion of Sbruumetinib, as shown in a phase II trial. However, these findings were not validated in subsequent phase III studies (73, 74). Hence, additional investigation is required to further examine the impact of Selumetinib on LUAD. Nilotinib, as a tyrosine kinase inhibitor, is a new type of targeted therapy drug for chronic myeloid leukemia (75). The combination of Nilotinib and PD-L1 inhibition can reverse T-cell dysfunction and effectively prevent relapse in cases of acute B-cell leukemia (76). JNK.9L as a JNK inhibitor affect tumors by regulating related pathways (77). The exact effects of JNK.9L and Nilotinib on LUAD have not been determined. There is still a need for further investigation in this regard. Additionally, these findings imply that utilizing DRPS for drug selection could potentially enhance precision medicine for LUAD.

## 5 Conclusion

In this study, we have provided novel insights into the role of disulfidptosis in LUAD by employing an integrated analysis of both scRNA-seq and bulk RNA-seq data. Our findings shed light on the intricate landscape of the tumor microenvironment and the inherent heterogeneity within LUAD. By delineating the differentiation trajectories of distinct endothelial cell subtypes within LUAD, we have deciphered the shifts in expression patterns of disulfidptosis-related genes throughout the course of malignant transformation. Leveraging the shared genes between endothelial cell differentially expressed genes and the disulfidptosis gene set, we have formulated a prognostic risk signature (DRPS), which holds the potential to offer multifaceted application in LUAD ranging from diagnosis, treatment, prognosis prediction, and association with immunotherapy response. The differential expressions of gene panel in DRPS may serve as diagnostic biomarkers for LUAD, providing more possibilities for early screen. Several key signaling pathways associated with the development of LUAD have been indicated via the DRPS, which may contribute to personalized therapy. The DRPS is also able to predict the prognosis of patients with LUAD, prioritizing clinicians to identify potential high-risk individuals, we further demonstrate that the DRPS can distinguish the individuals that are potentially sensitive to immunotherapy. Additionally, DRPS may provide target information for the exploitation of RNA interference, small molecule drug, and immunotherapy in LUAD.

## Data availability statement

The datasets presented in this study can be found in online repositories. The names of the repository/repositories and accession number(s) can be found in the article/supplementary material.

## Ethics statement

The manuscript presents research on animals that do not require ethical approval for their study.

## Author contributions

DH: Writing – original draft, Writing – review & editing. HT: Software, Writing – original draft. XY: Software, Formal Analysis, Writing – review & editing. XL: Methodology, Validation, Writing – review & editing. YZ: Methodology, Validation, Writing – review & editing. JS: Data curation, Supervision, Formal Analysis, Writing – original draft.
